# OLDER ADULTS AS RESOURCES IN EARLY CHILDHOOD EDUCATION

**DOI:** 10.1093/geroni/igae098.0560

**Published:** 2024-12-31

**Authors:** Claire Growney, Claire Jordan, Carol Larson, Laura Carstensen

**Affiliations:** Stanford University, Stanford, California, United States; Stanford University, Stanford, California, United States; Stanford University, Stanford, California, United States; Stanford University, Stanford University, California, United States

## Abstract

As California rolls out universal transitional kindergarten for 4-year-olds, the public school system faces a shortage of teachers and paraeducators to meet minimum adult–child ratios. Given age-related strengths in the social-emotional domain, older adults may be especially well-suited to support young children’s developmental needs. We are currently forming and researching programs that bring older adults into early childhood education classrooms to support children in various capacities. Many schools have used volunteers, but our focus is paid hourly and salaried positions. To inform program development, we collected qualitative and quantitative data from 8 early childhood center directors and 10 adults aged 55–73 who indicated interest in working with young children. Directors’ top-rated goals were to recruit people who would contribute to a calm, inviting classroom environment, support children’s social-emotional needs, and lower adult-child ratios. Directors also reported concerns, including older adults being unable to meet the physical demands of the job. Older adults’ top-rated reasons for being interested were wanting to have an emotionally meaningful experience and make a difference for young children. Information learned from these data was used to inform the creation of pilot programs at sites in Los Angeles, Fresno, and Santa Cruz, California. At baseline and the end of the programs, we are collecting assessments from classroom teachers, directors, and older adult participants to examine effects of the program on classroom environment, attitudes toward older adults, and older adults’ well-being. We will discuss barriers to program implementation and methods for successfully recruiting and retaining participants.

